# Left-atrial long-axis shortening allows effective quantification of atrial function and optimized risk prediction following acute myocardial infarction

**DOI:** 10.1093/ehjopen/oeac053

**Published:** 2022-08-12

**Authors:** Sören J Backhaus, Simon F Rösel, Thomas Stiermaier, Jonas Schmidt-Rimpler, Ruben Evertz, Alexander Schulz, Torben Lange, Johannes T Kowallick, Shelby Kutty, Boris Bigalke, Matthias Gutberlet, Gerd Hasenfuß, Holger Thiele, Ingo Eitel, Andreas Schuster

**Affiliations:** Department of Cardiology and Pneumology, University Medical Center Göttingen, Georg-August University, Robert-Koch-Str. 40, 37099 Göttingen, Germany; German Center for Cardiovascular Research (DZHK), partner site Göttingen, Göttingen, Germany; Department of Cardiology and Pneumology, University Medical Center Göttingen, Georg-August University, Robert-Koch-Str. 40, 37099 Göttingen, Germany; German Center for Cardiovascular Research (DZHK), partner site Göttingen, Göttingen, Germany; University Heart Center Lübeck, Medical Clinic II (Cardiology/Angiology/Intensive Care Medicine), University Hospital Schleswig-Holstein, Lübeck, Germany; Germany and German Center for Cardiovascular Research (DZHK), partner site Hamburg/Kiel/Lübeck, Lübeck, Germany; Department of Cardiology and Pneumology, University Medical Center Göttingen, Georg-August University, Robert-Koch-Str. 40, 37099 Göttingen, Germany; Department of Cardiology and Pneumology, University Medical Center Göttingen, Georg-August University, Robert-Koch-Str. 40, 37099 Göttingen, Germany; German Center for Cardiovascular Research (DZHK), partner site Göttingen, Göttingen, Germany; Department of Cardiology and Pneumology, University Medical Center Göttingen, Georg-August University, Robert-Koch-Str. 40, 37099 Göttingen, Germany; German Center for Cardiovascular Research (DZHK), partner site Göttingen, Göttingen, Germany; Department of Cardiology and Pneumology, University Medical Center Göttingen, Georg-August University, Robert-Koch-Str. 40, 37099 Göttingen, Germany; German Center for Cardiovascular Research (DZHK), partner site Göttingen, Göttingen, Germany; University Medical Center Göttingen, Institute for Diagnostic and Interventional Radiology, Georg-August University, Göttingen, Germany; Department of Pediatrics, The Blalock-Taussig-Thomas Pediatric and Congenital Heart Center, Johns Hopkins School of Medicine, Johns Hopkins University, 1800 Orleans St, Baltimore, MD 21287, USA; Department of Cardiology, Charité Campus Benjamin Franklin, University Medical Center Berlin, Berlin, Germany; Heart Center Leipzig, University of Leipzig, Institute of Diagnostic and Interventional Radiology, Leipzig, Germany; Department of Cardiology and Pneumology, University Medical Center Göttingen, Georg-August University, Robert-Koch-Str. 40, 37099 Göttingen, Germany; German Center for Cardiovascular Research (DZHK), partner site Göttingen, Göttingen, Germany; Department of Internal Medicine/Cardiology, Heart Center Leipzig at University of Leipzig, Leipzig, Germany; University Heart Center Lübeck, Medical Clinic II (Cardiology/Angiology/Intensive Care Medicine), University Hospital Schleswig-Holstein, Lübeck, Germany; Germany and German Center for Cardiovascular Research (DZHK), partner site Hamburg/Kiel/Lübeck, Lübeck, Germany; Department of Cardiology and Pneumology, University Medical Center Göttingen, Georg-August University, Robert-Koch-Str. 40, 37099 Göttingen, Germany; German Center for Cardiovascular Research (DZHK), partner site Göttingen, Göttingen, Germany

**Keywords:** Myocardial infarction, Left-atrial long-axis strain, Prognosis, Atrial physiology

## Abstract

**Aims:**

Deformation imaging enables optimized risk prediction following acute myocardial infarction (AMI). However, costly and time-consuming post processing has hindered widespread clinical implementation. Since manual left-ventricular long-axis strain (LV LAS) has been successfully proposed as a simple alternative for LV deformation imaging, we aimed at the validation of left-atrial (LA) LAS.

**Methods and results:**

The AIDA STEMI and TATORT-NSTEMI trials recruited 795 patients with ST-elevation myocardial infarction and 440 with non-ST-elevation myocardial infarction. LA LAS was assessed as the systolic distance change between the middle of a line connecting the origins of the mitral leaflets and either a perpendicular line towards the posterior atrial wall (LAS_90_) or a line connecting to the LA posterior portion of the greatest distance irrespective of a predefined angle (LAS). Primary endpoint was major adverse cardiac event (MACE) occurrence within 12 months. There were no significant differences between LA LAS and LAS_90_, both with excellent reproducibility. LA LAS correlated significantly with LA reservoir function (Es, *r* = 0.60, *P* < 0.001). Impaired LA LAS resulted in higher MACE occurrence [hazard ratio (HR) 0.85, 95% confidence interval (CI) 0.82–0.88, *P* < 0.001]. LA LAS (HR 0.90, 95% CI 0.83–0.97, *P* = 0.005) and LV global longitudinal strain (GLS, *P* = 0.025) were the only independent predictors for MACE in multivariate analyses. C-statistics demonstrated incremental value of LA LAS in addition to GLS (*P* = 0.016) and non-inferiority compared with FT Es (area under the receiver operating characteristic curve 0.74 vs. 0.69, *P* = 0.256).

**Conclusion:**

Left-atrial LAS provides fast and software-independent approximations of quantitative LA function with similar value for risk prediction compared with dedicated deformation imaging.

**Clinical trial registration:**

ClinicalTrials.gov: NCT00712101 and NCT01612312

## Graphical Abstract

**Graphical abstract:**
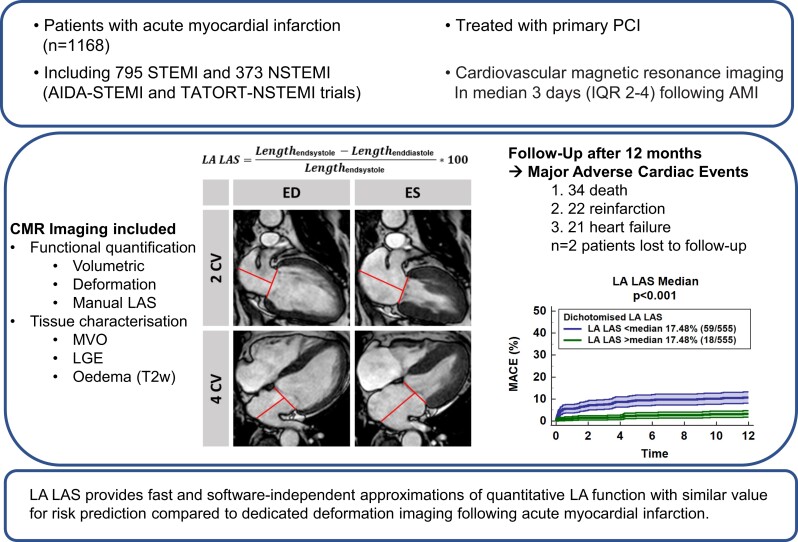

## Introduction

Coronary artery and ischaemic heart disease represent a substantial share of cardiovascular disease burden.^1^ Percutaneous coronary intervention (PCI)^2,3^ and a broad spectrum of drugs available for the treatment of heart failure^4^ have substantially improved the prognosis of ischaemic heart disease. Albeit its limitations, left-ventricular ejection fraction (LVEF) remains the reference standard^5^ for clinical decision-making on drug therapy (e.g. spironolactone)^4^ or preventive interventions such as implantable cardioverter-defibrillator (ICD) therapy^6^ in ischaemic heart failure. While the sole use of LVEF has already been challenged,^7^ myocardial deformation imaging has demonstrated improved sensitivity for the detection of myocardial dysfunction in the presence of preserved LVEF^8^ as well as superiority for the prediction of major adverse cardiac events (MACEs) in ischaemic and non-ischaemic heart disease.^9–11^ Notwithstanding, introduction of deformation imaging in clinical routine has been slowed down by costly post-processing and limited agreement between software solutions without uniform reference standards for strain assessment.^12^ Manual left-ventricular (LV) long-axis strain (LAS) has been introduced as a simple and reliable approximation of LV global function with similar predictive prognostic value when compared with LV global longitudinal strain (GLS)^13^ following acute myocardial infarction (AMI).^14^ This technique has also been successfully adopted to cardiac magnetic resonance (CMR) left-atrial (LA) longitudinal deformation assessment in a small heart failure cohort with preserved ejection fraction (HFpEF).^15^ The aim of the present study was thus the validation of this approach and the evaluation of its prognostic significance in a large population of ST-elevation myocardial infarction (STEMI) and non-STEMI (NSTEMI) patients.

## Methods

### Study population

Patients recruited to the AIDA STEMI (Abciximab Intracoronary vs. intravenously Drug Application in STEMI, NCT00712101)^16^ or TATORT-NSTEMI trials (Thrombus Aspiration in Thrombus Containing Culprit Lesions in Non-ST-Elevation, NCT01612312)^17^ could participate in an additional CMR substudy if eligible.^18^ Briefly, the AIDA STEMI trial enrolled 2065 STEMI patients for randomization to intracoronary (*n* = 1032) or intravenous (*n* = 1033) abciximab bolus application during PCI, 795 of which additionally underwent CMR imaging. The TATORT-NSTEMI trial enrolled 440 NSTEMI patients for randomization to aspiration thrombectomy (*n* = 221) or standard PCI (*n* = 219) followed by CMR for assessment of microvascular injury. The primary clinical endpoint of both CMR substudies was the occurrence of MACE consisting of all-cause mortality, reinfarction, and hospitalization due to congestive heart failure within 12 months after AMI. Clinical endpoints reported by each trial site were evaluated by a blinded endpoint committee. Each patient could account for one MACE only. In the occurrence of multiple events, the order of prioritization was first death, second reinfarction, and third congestive heart failure. The lead ethical committee at the University of Leipzig and local ethical committees at involved partner sites approved both studies as well as the CMR substudy. All patients gave written informed consent before randomization. The studies were conducted according to the principles of the Helsinki Declaration. The CMR substudy was supported by the German Centre for Cardiovascular Research (DZHK).

### Cardiovascular magnetic resonance imaging

**Figure 1:**
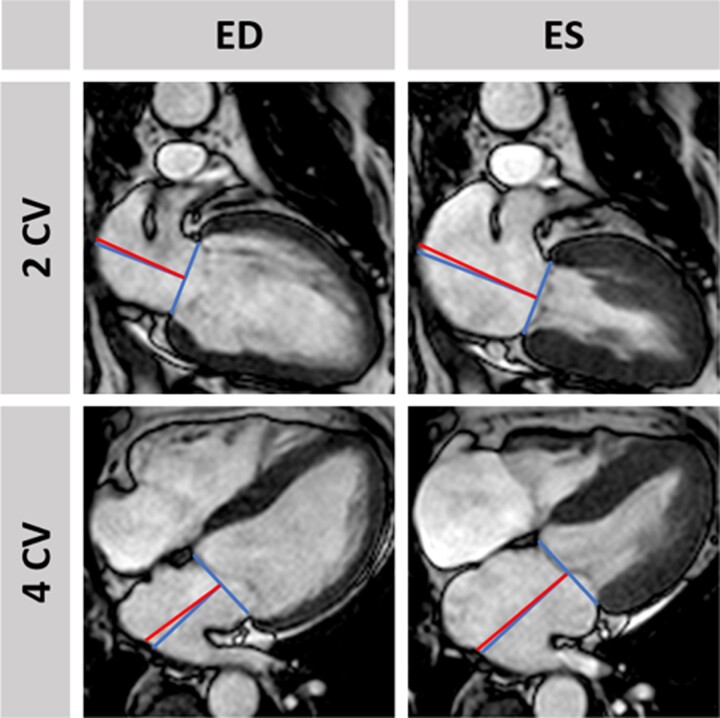
Left-atrial long-axis strain assessment. The figure illustrates the assessment of left-atrial long-axis strain in the two- and four-chamber view. It was assessed between the middle of a line connecting the origins of the mitral leaflets and either a perpendicular line towards the posterior atrial wall (left-atrial long-axis strain_90_, blue, perpendicular line) or a line connecting to the left-atrial posterior portion of the greatest distance in regards to the middle of the mitral reference line (left-atrial long-axis strain, red, non-perpendicular line).

An identical CMR imaging protocol was conducted across all study sites on clinical 1.5 or 3.0 Tesla scanners.^18^ The protocol included balanced steady-state free precession (bSSFP) long-axis two- and four-chamber views (CVs) and a short-axis (SA) stack, late gadolinium enhancement (LGE) for the evaluation of infarct size (IS), and microvascular obstruction (MO) as well as T_2_-weighted images for the assessment of the area at risk and myocardial salvage, respectively. Blinded CMR functional analyses were performed in a core-laboratory. The presence of mitral regurgitation was assessed visually on steady-state free precession long-axis cine sequences. LVEF was assessed in SA stacks, left-atrial volume index LAEF using a biplane approach, respectively LV/LA EF = ((vol_max_ − vol_min_)/vol_max_) × 100. LA LAS was assessed between the middle of a line connecting the origins of the mitral leaflets and either a perpendicular line towards the posterior atrial wall (LA LAS_90_) or a line connecting to the LA posterior portion of the greatest distance in regards to the middle of the mitral reference line (LA LAS, *Figure 1*). LA LAS was calculated as follows:

$$\mathrm{LA}\;\mathrm{LAS}=\frac{\mathrm{Lengt}h_{\mathrm{endsystole}}-\mathrm{Lengt}h_{\mathrm{enddiastole}}}{\mathrm{Lengt}h_{\mathrm{endsystole}}}\times 100$$

LA LAS values were based on the average of 2- and 4-CVs. CMR-FT was performed in identical bSSFP cine images using established and validated post-processing software (2D CPA MR, Cardiac Performance Analysis, Version 1.1.2; TomTec Imaging Systems, Unterschleissheim, Germany^19,20^) as previously described^21^ for the evaluation of LV GLS and LA reservoir function (Es). Briefly, LV and LA endocardial contours were manually traced in end-diastole on 2- and 4-CVs. Subsequently, the software algorithm was applied following image features throughout the whole cardiac cycle. The contours were manually reviewed, and corrections were made to the manual end-diastolic contours only. Peak LV/LA strain values were taken from the plotted strain curve of the cardiac cycle. Final values were calculated based on the average of three independently repeated measurements.

### Statistical analyses

Categorical variables are reported as frequencies and corresponding percentages. Differences were tested using the χ^2^. Continuous variables are reported as median with associated interquartile ranges (IQRs) after testing for normal distribution using the Shapiro–Wilk test. Comparisons were performed using the non-parametric Mann–Whitney *U* test. Correlations were assessed by the means of Spearman rank correlation coefficients. Predictors for predefined endpoints were identified from uni- and multivariable Cox regression analyses reported as hazard ratios (HRs) with 95% confidence intervals (CIs) for continuously tested variables, Kaplan–Meier plots with associated log-rank test, as well as area under the receiver operating characteristic curve (AUC) analyses. AUC comparisons were performed using the method proposed by DeLong *et al*.^22^ Intra- and interobserver reproducibility was assessed in 40 patients, including 20 randomly selected patients from the STEMI and NSTEMI collective. Reproducibility calculations comprised mean differences (MDs) and their standard deviation (SD) as well as intraclass correlation coefficients (ICCs) and coefficients of variation (CoV) defined as the SD of the differences divided by the mean. The level of agreement was considered excellent for ICC > 0.90, good for 0.90–0.75, moderate for 0.5–0.75, and poor for <0.5.^23^ Statistical calculations were performed using IBM SPSS Statistic Software Version 26 for Windows (IBM, Armonk, NY, USA) and MedCalc version 18.2.1 (MedCalc Software bvba, Ostend, Belgium). A two-tailed *P*-value <0.05 was considered statistically significant.

## Results

### Study population

**Figure 2:**
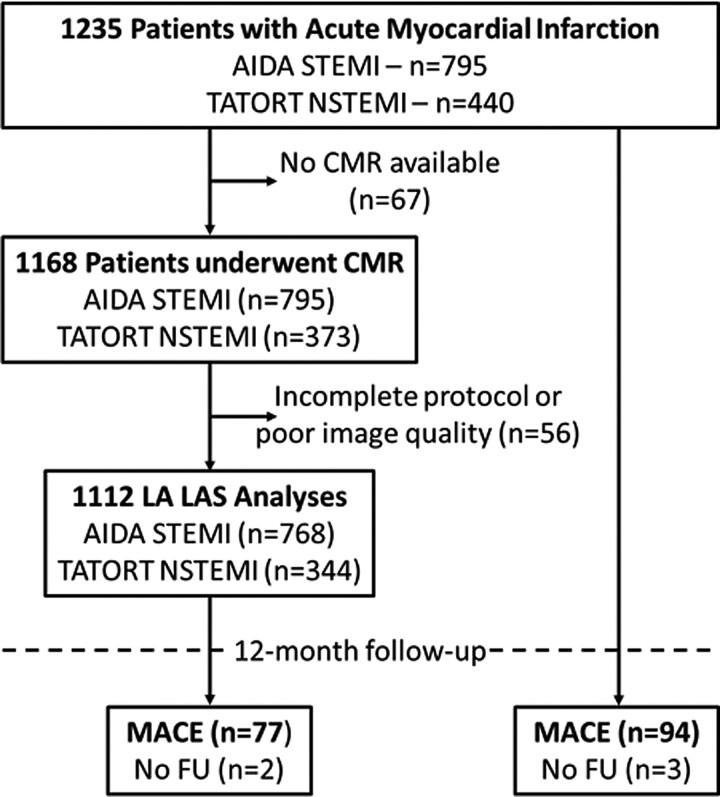
Study flow chart.

Of the 1235 initially enrolled patients (795 STEMI and 440 NSTEMI), 67 did not undergo CMR imaging and an additional 56 patients were excluded due to either incomplete or insufficient CMR image quality for post processing. Around 1112 complete data sets of 760 STEMI and 347 NSTEMI patients entered the final analysis (*Figure 2*). Baseline characteristics are reported in *Table 1*. CMR imaging was conducted in median 3 days (IQR 2–4) following AMI, 77 MACE were recorded during the 12 months follow-up period (STEMI: death: *n* = 20, reinfarction *n* = 18, HF admission *n* = 14; NSTEMI: death: *n* = 14, reinfarction *n* = 4, HF admission *n* = 7). Statistically most distinct differences in patients with MACE compared with patients without were older age (*P* < 0.001), higher Killip class on admission (*P* = 0.001) as well as a higher number of diseased coronary vessels (*P* = 0.009). CMR-derived infarct characteristics are reported in *Table 2*. Patients with MACE during follow up had significantly larger IS (20.4 vs. 13.1%, *P* = 0.001) and MO (0.88 vs. 0.33%, *P* = 0.041) with a strong trend for a larger area at risk (33.6 vs. 29.3%, *P* = 0.068). The myocardial salvage index (44.5 vs. 55.5, *P* = 0.024) was significantly smaller in patients with MACE during follow up.

**Table 1:** Baseline characteristics

Variable	All patients *n* = 1112	MACE *n* = 77	No MACE *n* = 1033	*P-*value
*Cardiovascular risk factors*				
Age (years)	64 (53–72)	72 (61–77)	63 (52–72)	**<0**.**001**
Male sex	837/1112 (75.3)	51/77 (66.2)	785/1033 (76.0)	0.055
Active smoking	449/1032 (43.5)	21/70 (30.0)	427/960 (44.5)	**0**.**018**
Hypertension	792/1110 (71.4)	64/77 (83.1)	726/1031 (70.4)	0.017
Hyperlipoproteinaemia	421/1104 (38.1)	26/77 (33.8)	394/1025 (38.4)	0.416
Diabetes	260/1110 (23.6)	27/77 (35.1)	232/1031 (22.5)	**0**.**012**
Body mass index (kg/m²)	27.5 (25.0–30.4)	27.3 (25.3–31.1)	27.5 (24.9–30.3)	0.601
Previous myocardial infarction	76/1110 (6.8)	5/77 (6.5)	70/1031 (6.8)	0.921
ST-segment elevation	768/1112 (69.1)	52/77 (67.5)	716/1033 (69.3)	0.744
Time symptoms to balloon^a^ (min)	180 (109–315)	192 (116–373)	180 (108–310)	0.306
Atrial fibrillation	64/1108 (5.8)	11/77 (14.3)	53/1029 (5.2)	**0**.**001**
Mitral regurgitation	571/1077 (53.0)	54/75 (72.0)	564/1002 (56.3)	**0**.**024**
*Killip class on admission*				**<0.001**
1	985/1112 (88.6)	50/77 (64.9)	933/1033 (90.3)	
2	88/1112 (7.9)	18/77 (23.4)	70/1033 (6.8)	
3	23/1112 (2.1)	5/77 (6.5)	18/1033 (1.7)	
4	16/1112 (1.4)	4/77 (5.2)	12/1033 (1.2)	
*Diseased vessels*				**0.009**
1	555/1112 (49.9)	28/77 (36.4)	526/1033 (50.9)	
2	333/1112 (29.9)	24/77 (31.2)	309/1033 (29.9)	
3	224/1112 (20.1)	25/77 (32.5)	198/1033 (19.2)	
*Affected artery*				0.140
Left anterior descending	454/1112 (40.8)	41/77 (53.2)	413/1033 (40.0)	
Left circumflex	233/1112 (21.0)	15/77 (19.5)	216/1033 (20.9)	
Left main	5/1112 (0.4)	0/77 (0.0)	5/1033 (0.5)	
Right coronary artery	413/1112 (37.1)	20/77 (26.0)	393/1033 (38.0)	
Bypass graft	7/1112 (0.6)	1/77 (1.3)	6/1033 (0.6)	
*TIMI flow grade before PCI*				0.558
0	558/1112 (50.2)	44/77 (57.1)	513/1033 (49.7)	
1	129/1112 (11.6)	6/77 (7.8)	123/1033 (11.9)	
2	226/1112 (20.3)	14/77 (18.2)	211/1033 (20.4)	
3	199/1112 (17.9)	13/77 (16.9)	186/1033 (18.0)	
Stent implanted	1085/1112 (97.6)	75/77 (97.4)	1008/1033 (97.6)	0.636
*TIMI flow grade after PCI*				0.154
0	21/1112 (1.9)	1/77 (1.3)	20/1033 (1.9)	
1	23/1112 (2.1)	4/77 (5.2)	19/1033 (1.8)	
2	83/1112 (7.5)	8/77 (10.4)	75/1033 (7.3)	
3	985/1112 (88.6)	64/77 (83.1)	919/1033 (89.0)	
Time to CMR (days)	3 (2–4)	3 (2–4)	3 (2–4)	**0**.**024**

Data presented as *n*/*N* (%) or median (IQR). *P*-values were calculated for the comparison between patients with and without MACE, continuous variables were tested using the Mann–Whitney *U* test, categorical variables were tested using the χ^2^. Numbers in bold indicate statistical significance.

CABG, coronary artery bypass graft; MACE, major adverse cardiac event; PCI, percutaneous coronary intervention; TIMI, thrombolysis in myocardial infarction; CMR, cardiovascular magnetic resonance.

a Only assessed in STEMI patients (*n* = 768).

**Table 2:** Cardiac magnetic resonance–derived morphological and functional infarct characterization

Variable	All patients	MACE	No MACE	*P*-value
*Myocardial infarction*				
Infarct size (%)	13.4 (5.4–21.8)	20.4 (9.8–29.0)	13.1 (5.3–21.4)	**0**.**001**
Microvascular obstruction (%)	0.36 (0.00–2.00)	0.88 (0.00–2.86)	0.33 (0.00–1.93)	**0**.**041**
Area at risk (%)	29.4 (20.3–42.6)	33.6 (24.2–45.9)	29.3 (20.3–42.4)	0.068
Myocardial salvage index	54.7 (33.7–74.7)	44.5 (23.4–69.1)	55.5 (35.0–74.7)	**0**.**024**
*Ventricular function*				
LVEF (%)	50.6 (43.5–57.6)	40.0 (33.1–52.4)	51.0 (44.3–57.6)	**<0**.**001**
LV GLS (%)	–16.4 (–12.4, –20.1)	–11.6 (–8.3, –17.1)	–16.6 (–12.9, –20.3)	**<0**.**001**
*Atrial function*				
LA Es (%)	20.9 (16.3–25.8)	16.2 (11.6–21.3)	21.2 (16.7–26.2)	**<0**.**001**
LAEF (%)	53.3 (46.5–59.3)	44.2 (35.2–52.0)	53.8 (47.1–59.5)	**<0**.**001**
LA LAS (%)	17.5 (14.5–20.6)	12.7 (9.3–17.4)	17.7 (14.8–20.8)	**<0**.**001**
LA LAS_90_ (%)	19.9 (16.1–23.6)	14.7 (10.6–19.0)	20.2 (16.6–23.9)	**<0**.**001**

Data presented as median with associated interquartile range. *P*-values were calculated for the comparison between patients with and without MACE using the Mann–Whitney *U* test. Numbers in bold indicate statistical significance.

LV, left-ventricle; EF, ejection fraction; GLS, global longitudinal strain; LA, left atrium; Es, reservoir function; LAS, long-axis strain.

### Cardiac functional evaluation

Cardiac functional parameters are reported in *Table 2*. Both LV function as assessed by LVEF (40.0 vs. 51.0%) and GLS (−11.6 vs. −16.6%) as well as LA function by LA Es (16.2 vs. 21.2%), LAEF (44.2 vs. 53.8%), and LAS/LAS_90_ (12.7/14.7 vs. 17.7/20.2%) were distinctly impaired in patients with MACE during follow up (*P* < 0.001 for all).

LA LAS (median 17.5%, IQR 14.5–20.6) was significantly lower compared with LA LAS_90_ (median 19.9%, IQR 16.1–23.6) which was significantly lower compared with LA Es (*P* < 0.001 for all). LA LAS and LA LAS_90_ correlated significantly with each other (*r* = 0.88, *P* < 0.001) as well as with LA Es (*r* = 0.60 and 0.62, *P* < 0.001) and LAEF (*r* = 0.65 and 0.66, *P* < 0.001). While LA LAS could be evaluated in 1112 patients, LA Es was more prone to impaired image quality and artefacts resulting in a total of 1044 complete data sets. Reproducibility of LA LAS (intraobserver: ICC 0.94, CoV 12.2%, MD/SD 0.68/2.17; interobserver: ICC 0.91, CoV 9.4%, MD/SD 0.92/1.84) as well as LA LAS_90_ (intraobserver: ICC 0.96, CoV 15.3%, MD/SD 1.00/2.75; interobserver: ICC 0.91, CoV 15.6%, MD/SD 1.42/3.11) was excellent. Bland–Altmann plots are shown in Supplementary material online, *Figure S1*. The complete analysis of one patient’s data set using LA LAS took in general below 2 min.

### Outcome

**Figure 3:**
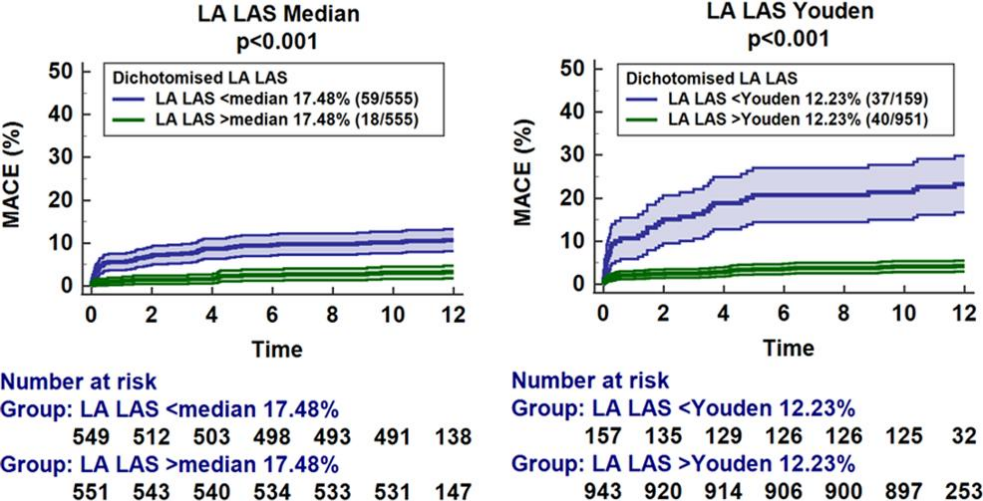
Left-atrial strain for major adverse cardiac event prediction. The graphs show the association of left-atrial long-axis strain dichotomized at the median of 17.48% as well as according to the Youden index at 12.23% on the rate of major adverse clinical events including associated 95% confidence intervals as well as the patients’ number at risk.

**Figure 4:**
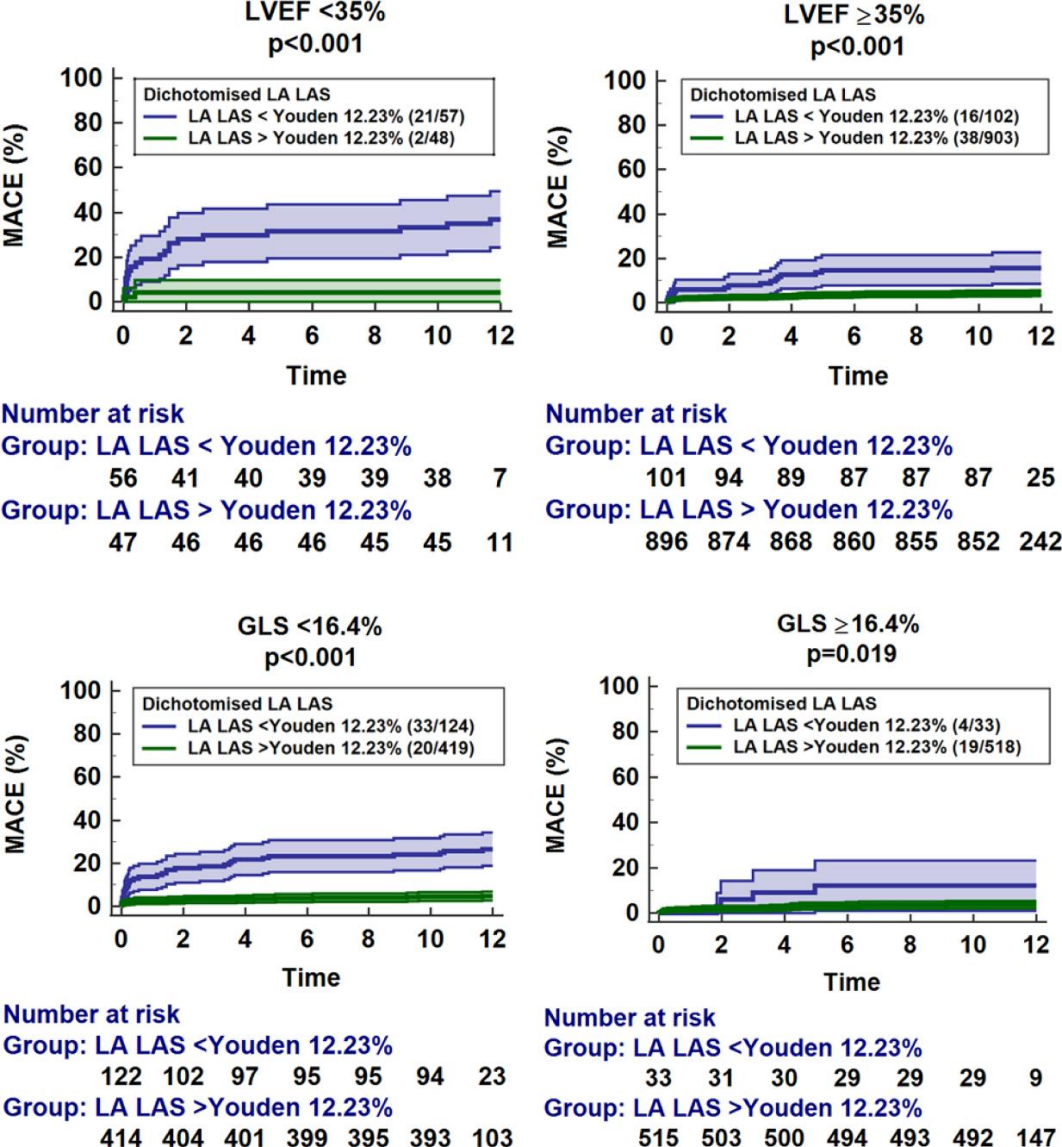
Incremental value of left-atrial long-axis strain. The graphs demonstrate the impact of additional left-atrial long-axis strain analysis over (top) left-ventricular ejection fraction above and below a cut-off at 35% as well as (bottom) left-ventricular global longitudinal strain above or below the median of 16.4%. The cut-off for left-atrial long-axis strain was identified with the Youden index at 12.23%. The graph shows the rate of major adverse clinical events including associated 95% confidence intervals as well as the patients’ number at risk.

Uni- and multivariate Cox regression analyses are reported in *Tables 3 and 4* as well as Supplementary material online, *Tables S1–S5*. There was no difference between LA LAS und LA LAS_90_ and consequently results are reported for LA LAS only. Univariate Cox regression analyses revealed LA LAS as a highly significant predictor of MACE within CMR-derived functional parameters (HR 0.85, 95% CI 0.82–0.88, *P* < 0.001). After correction for all univariate significant parameters (LAEF, LA Es, LA LAS90 and LA LAS were not tested in the same multivariate model due to high colinearity), only LA LAS (HR 0.90, 95% CI 0.82–0.99, *P* = 0.036) and LV GLS (HR 1.10, 95% CI 1.02–1.20, *P* = 0.015) remained independent predictors for MACE occurrence during 12 months follow up (*Table 4*). The prognostic value of LA LAS was independent of the presence of atrial fibrillation (HR 0.85, 95% CI 0.81–0.89, *P* < 0.001). The presence of MR was associated with MACE in univariate (HR = 2.18, 95% CI 1.18–3.91, *P* = 0.010) analyses but not independently of LA LAS (HR 1.56, 95% CI 0.86–2.84, *P* = 0.144) in a multivariate model. Results for STEMI and NSTEMI subgroups are shown in Supplementary material online, *Tables S1–S4*. Considering fewer variables in the multivariate model to address overfitting further underlines the impact of LA LAS (HR 0.91, 95% CI 0.85–0.98, *P* = 0.007) independent of GLS and Killip class (Supplementary material online, *Table S5*). Discrimination of low- and high-risk groups for MACE using either the median of LA LAS or dichotomization according to the Youden index is demonstrated by Kaplan–Meier curves (*P* < 0.001, *Figure 3*). Furthermore, the LA LAS dichotomized by the means of the Youden index at 12.23% discriminated low- and high-risk groups in patients with mildly to moderately (≥35%, *P* < 0.001) as well as highly impaired (<35%, *P* < 0.001) LVEF or LV GLS above (*P* = 0.019) and below (*P* < 0.001) the median (−16.4%; *Figure 4*).

**Table 3:** Predictors of major adverse cardiac event in univariate Cox regression analysis

Variable	Univariable hazard ratio (CI)	*P*-value
*Cardiovascular/clinical risk factors*		
Age	1.05 (1.03–1.07)	**<0**.**001**
Male Sex	0.63 (0.39–1.02)	0.058
Smoking	0.55 (0.33–0.92)	**0**.**022**
Hypertension	2.03 (1.12–3.68)	**0**.**020**
HLP	0.83 (0.52–1.33)	0.430
Diabetes	1.82 (1.14–2.90)	**0**.**012**
BMI	1.02 (0.97–1.07)	0.479
Killip class	2.05 (1.62–2.59)	**<0**.**001**
Atrial fibrillation	2.83 (1.49–5.35)	**0**.**001**
Mitral regurgitation	2.18 (1.21–3.91)	**0**.**010**
*Angiography*		
Diseased vessels	1.50 (1.15–1.97)	**0**.**004**
Culprit lesion	1.37 (1.10–1.72)	**0**.**006**
TIMI pre	0.92 (0.76–1.11)	0.379
TIMI post	0.82 (0.59–1.15)	0.244
*CMR-derived morphology*		
IS	1.03 (1.01–1.05)	**<0**.**001**
MVO	1.08 (1.02–1.15)	**0**.**005**
AAR	1.01 (1.00–1.03)	0.120
MSI	0.99 (0.98–1.00)	**0**.**034**
LAVI	1.03 (1.02–1.04)	**<0**.**001**
*CMR-derived function*		
LVEF	0.94 (0.92–0.96)	**<0**.**001**
LV GLS	1.14 (1.09–1.19)	**<0**.**001**
LA Es	0.90 (0.87–0.94)	**<0**.**001**
LAEF	0.94 (0.93–0.96)	**<0**.**001**
LA LAS	0.85 (0.82–0.88)	**<0**.**001**
LA LAS_90_	0.86 (0.83–0.90)	**<0**.**001**

The table reports univariable Cox regression models to predict a major adverse clinical event during the 12 months follow-up period following acute myocardial infarction. Data are presented as hazard ratios with associated 95% confidence intervals in parentheses. Numbers in bold indicate statistical significance.

CI, confidence interval; HLP, hyperlipoproteinaemia; BMI, body mass index; TIMI, thrombolysis in myocardial infarction grade pre/post PCI; PCI, percutaneous coronary intervention; IS, infarct size; MVO, microvascular obstruction; AAR, area at risk; MSI, myocardial salvage index; LAVI, left-atrial volume index; LVEF, left-ventricular ejection fraction; GLS, global longitudinal strain; Es, reservoir function; LAS, long-axis strain.

**Table 4:** Multivariate predictors of major adverse cardiac event

Variable	1. Multivariate hazard ratio (CI)	2. Multivariate hazard ratio (CI)	3. Multivariate hazard ratio (CI)	4. multivariate hazard ratio (CI)
*Cardiovascular/clinical risk factors*				
Age				
Smoking				
Hypertension				
Diabetes				
Killip class				
Atrial fibrillation				
*Angiography*				
Diseased vessels				
Culprit lesion				
*CMR-derived morphology*				
IS				
MVO				
MSI				
LAVI				
*CMR-derived function* ^a^				
LVEF				
LV GLS	1.11 (1.02–1.20) *P* = 0.013	1.11 (1.02–1.20) *P* = 0.011	1.10 (1.02–1.20) *P* = 0.015	1.11 (1.03–1.20) *P* = 0.010
1 LA Es				
2 LAEF		0.96 (0.92–0.99) *P* = 0.017		
3 LA LAS			0.90 (0.82–0.99) *P* = 0.036	
4 LA LAS_90_				0.92 (0.84–1.00) *P* = 0.050

The table reports multivariable Cox regression models (based on the enter method) to predict a major adverse clinical event during the 12 months follow-up period following acute myocardial infarction. Data are presented as hazard ratios with associated 95% confidence intervals in parentheses. Variables with univariate significance (*P* < 0.05) were included in multivariable Cox regression models and are presented if they emerged as statistically significant (*P* < 0.05).

CI, confidence interval; HLP, hyperlipoproteinaemia; BMI, body mass index; IS, infarct size; MVO, microvascular obstruction; MSI, myocardial salvage index; LVEF, left-ventricular ejection fraction; LAVI, left-atrial volume index; GLS, global longitudinal strain; Es, reservoir function; LAS, long-axis strain.

a LA Es, LAEF, and LAS/LAS_90_ were considered in separate multivariate models due to their high correlation (Models 1–4).

Prognostic accuracy as assessed by AUC analyses is reported in *Table 5*. LA LAS emerged as the parameter with the numerically highest AUC (0.74, 95% CI 0.68–0.80) for the prediction of MACE and was not inferior for MACE prediction compared with FT Es (AUC 0.69, *P* = 0.256). The addition of LA LAS to LVEF (*P* = 0.011), GLS (*P* = 0.016), or MO/IS (*P* = 0.004) was superior for MACE prediction compared with these parameters on their own.

**Table 5:** C-statistics for major adverse cardiac event and mortality

Variable	AUC	Confidence interval	ROC comparison
*MACE*			
LVEF	0.69	0.61–0.76	
LA LAS	0.74	0.68–0.80	0.092
LVEF + LA LAS	0.74	0.68–0.81	**0**.**011**
MO + IS	0.65	0.58–0.73	
LA LAS	0.74	0.68–0.80	**0**.**011**
MO + IS + LAS	0.76	0.69–0.83	**0**.**004**
GLS	0.69	0.63–0.76	
LA LAS	0.74	0.68–0.80	0.247
GLS + LAS	0.75	0.69–0.81	**0**.**016**
*Mortality*			
LVEF	0.66	0.54–0.78	
LA LAS	0.77	0.68–0.85	**0**.**030**
LVEF + LA LAS	0.76	0.68–0.85	**0**.**018**
MO + IS	0.62	0.49–0.74	
LA LAS	0.77	0.68–0.85	**0**.**002**
MO + IS + LAS	0.81	0.71–0.90	**0**.**002**
GLS	0.73	0.63–0.83	
LAS	0.77	0.68–0.85	0.568
GLS + LAS	0.79	0.70–0.87	0.141

*P*-values were calculated for the AUC comparisons of left-atrial (LA) long-axis strain (LAS) compared with as well as in addition to the following parameters: Left-ventricular ejection fraction (LVEF), microvascular obstruction (MO), infarct size (IS), as well as global longitudinal strain (GLS). Calculations were performed using the method proposed by de Long *et al*. for MACE and mortality occurrence separately. Numbers in bold indicate a statistical significance.

## Discussion

The results from the present CMR substudy of the AIDA STEMI and TATORT-NSTEMI trials demonstrate feasibility and clinical value of LA LAS assessments in a large prospectively recruited patient cohort undergoing CMR following AMI. Impaired LA LAS is independently associated with MACE occurrence following AMI when related to classical risk factors. LA LAS assessments also emerged as non-inferior to FT deformation imaging parameters of both atrial and ventricular function. LA LAS offers a fast, reliable as well as software- and vendor-independent approach for atrial functional quantification suitable for easy clinical routine implementation.

There are many relevant CMR parameters to predict outcome following AMI.^24^ Volume-derived LVEF is still the most established calculation in clinical routine. Impaired LVEF is distinctly associated with sudden cardiac death (SCD) and absolute risk for SCD increases as LVEF decreases. However, what may be referred to as the prevention paradox, the majority of patients with SCD were attributed to the low-risk group because of its larger size, albeit lower individual risks.^25^ Notwithstanding LVEF <35% is used as the threshold for ICD implantation^6^ despite more than two-thirds of patients following AMI having an LVEF of 35% and above.^26^ The addition of LA LAS to LVEF offered incremental prognostic value for the prediction of MACE. Consequently, the singular use of LVEF may be challenged for adequate risk stratification for SCD in cardiovascular disease.^7^ Indeed, the sheer existence of HFpEF^8^ acknowledges the need for more precise assessments of cardiac pathophysiology in heart failure for improved risk stratification beyond volumetric analyses.

Cardiac magnetic resonance enables risk assessment for SCD in non-ischaemic and ischaemic cardiomyopathy by quantification of scar/fibrosis tissue^27^ as well as peri-infarct zone characterization as an arrhythmogenic substrate.^28,29^ The latter may also allow the prediction of appropriate ICD shocks.^30^ Assessment of IS and MO by LGE imaging enables precise risk stratification following AMI.^24^ To date, deformation imaging has been added to the product range of CMR. LV GLS demonstrated superiority in risk assessment following AMI in addition to either LVEF or IS.^10^ Similar results were found for LV LAS as a simple and fast approximation of LV function.^14^ Noteworthy, LA LAS emerged as an independent predictor for MACE in addition to LV GLS. Furthermore, it offered incremental value for risk prediction as appreciated from c-statistics and Kaplan–Meier curves compared with LV function (LVEF and LV GLS) and LV myocardial characterization (IS and MO).

Indeed, beyond the value of LV function, the role of atrial function has come to the fore^21^addressing on the one hand intrinsic atrial dysfunction^31^ and on the other hand atrial impairment as a reflection of ventricular disease.^15^ FT deformation imaging allows post processing of routinely acquired CMR cine sequences.^32^ FT enables the assessment of the three atrial functional phases reservoir, conduit, and booster pump function.^19^ Their detailed assessment allows the differentiation of atrial mechanics, pathophysiological changes, and compensatory mechanisms. Onset of diastolic dysfunction precedes systolic failure during the ischaemic cascade.^33^ Following STEMI, a compensatory increase of atrial active contractility for deteriorated passive conduit function associated with LV diastolic dysfunction has been described.^34^ The loss of which might indicate disease severity and is strongly associated with MACE following AMI.^21^ However, the value of atrial functional alterations lies beyond the link to LV function, and passive conduit function is associated with exercise capability in HFpEF independently of LV stiffness and relaxation.^35^ Despite incremental diagnostic and prognostic value, clinical implementation of deformation imaging has been complicated by costly post processing, limited intervendor agreement and undisclosed technical properties.^12,36^ While the importance of LA function following myocardial infarction has repeatedly been demonstrated,^21,37,38^ the significance of longitudinal shortening approximation using LA LAS had yet to be defined. Importantly, the results of the present study demonstrate that LA LAS as approximation of LA function is equally potent as dedicated deformation imaging for risk assessment following myocardial infarction. While reproducibility of LA LAS was similar compared with FT Es,^21^ LA LAS is universally available and cheaper and not prone to intervendor variability.

Since LV LAS only represents an approximation of LV function, it does not provide information on strain rates and regional myocardial deformation. Notwithstanding, LV LAS has been successfully demonstrated as a reliable surrogate of LV deformation with similar prognostic value compared with dedicated deformation imaging^14^ while representing a fast, easy, and software-independent alternative.^13^ Furthermore, the loss of information caused by simple measurement of LV length might increase reliability being less susceptible to through-plane motion.^36^ In the present study, we validated a similar approach for approximation of LA LAS. Two methods were applied, one relying on observer discretion to visually define the long axis and identifying the correct distal LA posterior portion, and the second method thought to compare a predefined approach by a standing definition of long-axis measurement perpendicular to a line connecting the origins of the mitral leaflets. While the perpendicular approach benefits from a clear definition, it is prone to anatomic variations of the atrium which might need adjustments of the longitudinal direction. Since they were similar in reproducibility and diagnostic accuracy, LA LAS emerges as a reliable parameter for the approximation of LA longitudinal shortening.

### Limitations

Cardiac magnetic resonance imaging was conducted on scanners from different vendors with field strengths of 1.5 or 3.0 Tesla. However, all CMR systems are clinically well established and performed a standardized protocol, and CMR deformation imaging has shown to be reproducible independent of field strength.^39^ On the one hand, a selection bias may have occurred with unstable patients following AMI being excluded from CMR imaging, and on the other hand, preserved statistical significance in stable patients with lower event rates indicates reliability of the test. Conclusions on simple manual software-independent atrial longitudinal functional assessment rely on LA LAS assessment only, while MAPSE has not been assessed in this population. A penalized statistical model based on shrinkage has not been employed.^40^ The presence of mitral regurgitation was evaluated on bSSFP cine sequences only without severity grading.

## Conclusion

Left-atrial LAS provides fast and software-independent approximations of quantitative LA function. LA LAS is non-inferior for MACE prediction compared with dedicated FT deformation imaging approaches and an independent parameter for risk stratification. Consequently, it combines clinical feasibility with high accuracy and reproducibility available for the introduction to clinical routine assessments.

## Lead author biography


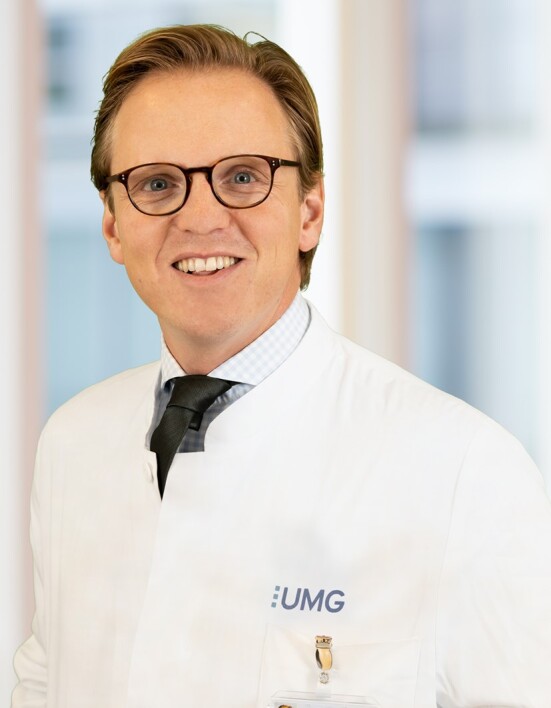
Prof. Andreas Schuster is an interventional cardiologist and cardiovascular imaging specialist. He graduated from Leipzig University Germany and received his cardiology training in Germany (Charite Hospital Berlin and University Medical Center Göttingen) and the UK (St Thomas’ Hospital London). He obtained research degrees from Leipzig University (MD research) and King’s College London, UK (PhD). He has a strong clinical and research interest in intervention and imaging and completed fellowships in advanced imaging at King’s College London (St Thomas’ Hospital) and interventional cardiology at the University of Sydney, Australia (Royal North Shore Hospital). He is currently professor of medicine and cardiology at the University Medical Center Göttingen where he also heads Cardiac Imaging and the Catheter Laboratories. He is fellow of the ESC, ACC, AHA, SCMR and SCCT and visiting professor at King's College London.

## Supplementary material

Supplementary material is available at *European Heart Journal Open* online.

## Supplementary Material

oeac053_Supplementary_Data

## Data Availability

Regarding data availability, we confirm that all relevant data are within the paper and all data underlying the findings are fully available without restriction from the corresponding author at the University Medical Centre Göttingen for researchers who meet the criteria for access to confidential data.
