# Supplementary material for: Left-atrial long-axis shortening allows effective quantification of atrial function and optimized risk prediction following acute myocardial infarction
Source: Eur Heart J Open. 2022 Aug 12;2(5):oeac053. doi: 10.1093/ehjopen/oeac053 (PMC9574426; doi:10.1093/ehjopen/oeac053)
Supplement: oeac053_Supplementary_Data [file oeac053_supplementary_data.docx]

**Supplements**

STEMI

| **Table S1 Univariate Predictors of MACE in STEMI** | | |
| --- | --- | --- |
| Variable | Univariable  Hazard Ratio (CI) | p |
| **Cardiovascular/clinical risk factors** | | |
| Age | 1.05 (1.03-1.08) | **<0.001** |
| Male Sex | 0.51 (0.29-0.79) | **0.017** |
| Smoking | 0.46 (0.24-0.88) | **0.019** |
| Hypertension | 2.70 (1.27-5.72) | **0.020** |
| HLP | 0.69 (0.38-1.23) | 0.209 |
| Diabetes | 1.92 (1.08-3.42) | **0.026** |
| BMI | 0.98 (0.92-1.05) | 0.599 |
| Killip-Class | 1.90 (1.46-2.49) | **<0.001** |
| Atrial fibrillation | 2.87 (1.35-6.10) | **0.006** |
| Mitral regurgitation | 2.00 (1.02-3.93) | **0.045** |
| **Angiography** | | |
| Diseased Vessels | 1.45 (1.04-2.00) | **0.027** |
| Culprit lesion | 1.17 (0.82-1.67) | 0.391 |
| TIMI pre | 0.81 (0.62-1.06) | 0.123 |
| TIMI post | 0.77 (0.52–1.15) | 0.201 |
| **CMR-derived morphology** | | |
| IS | 1.04 (1.02-1.06) | **<0.001** |
| MVO | 1.09 (1.02-1.16) | **0.008** |
| AAR | 1.02 (1.00-1.03) | 0.075 |
| MSI | 0.99 (0.97-1.00) | **0.020** |
| LAVI | 1.02 (1.01-1.04) | **0.007** |
| **CMR-derived function** | | |
| LV EF | 0.93 (0.91-0.96) | **<0.001** |
| LV GLS | 1.14 (1.08-1.20) | **<0.001** |
| LA Es | 0.90 (0.86-0.94) | **<0.001** |
| LA EF | 0.94 (0.92-0.96) | **<0.001** |
| LA LAS | 0.84 (0.80-0.88) | **<0.001** |
| LA LAS_90_ | 0.86 (0.83-0.90) | **<0.001** |
| The table reports univariable Cox regression models to predict a major adverse clinical event during the 12 months follow-up period following acute myocardial infarction in STEMI patients. The data is presented as hazard ratios with associated 95% confidence intervals in parentheses. CI = confidence interval, HLP =Hyperlipoproteinemia, BMI = body mass index, TIMI = Thrombolysis In Myocardial Infarction grade pre/post PCI, PCI = percutaneous coronary intervention, IS = infarct size, MVO = microvascular obstruction, AAR = area at risk, MSI = myocardial salvage index, LAVI = left atrial volume index, LV EF = left ventricular ejection fraction, GLS = global longitudinal strain, Es = reservoir function, LAS = long axis strain. | | |

| **Table S2 Multivariate Predictors of MACE in STEMI** | | | | |
| --- | --- | --- | --- | --- |
| Variable | 1. Multivariate Hazard Ratio (CI) | 2. Multivariate Hazard Ratio (CI) | 3. Multivariate Hazard Ratio (CI) | 4. Multivariate Hazard Ratio (CI) |
| **Cardiovascular/clinical risk factors** | | | | |
| Age |  |  |  |  |
| Male Sex |  |  |  |  |
| Smoking |  |  |  |  |
| Hypertension |  |  |  |  |
| Diabetes |  |  |  |  |
| Killip-Class |  |  |  |  |
| Atrial fibrillation |  |  |  |  |
| **Angiography** | | | | |
| Diseased Vessels |  |  |  |  |
| **CMR-derived morphology** | | | | |
| IS |  |  |  |  |
| MVO |  |  |  |  |
| MSI |  |  |  |  |
| LAVI |  |  |  |  |
| **CMR-derived function** | | | | |
| LV EF |  |  |  |  |
| LV GLS |  |  |  |  |
| 1 LA Es | 0.93 (0.87-0.99)  p=0.023 |  |  |  |
| 2 LA EF |  | 0.93 (0.89-0.97)  p<0.001 |  |  |
| 3 LA LAS |  |  | 0.90 (0.81-0.99)  p=0.037 |  |
| 4 LA LAS_90_ |  |  |  |  |
| The table reports multivariable Cox regression models (based on the enter method) to predict a major adverse clinical event during the 12 months follow-up period following acute myocardial infarction in STEMI patients. The data is presented as hazard ratios with associated 95% confidence intervals in parentheses. Variables with univariate significance (p<0.05) were included in multivariable Cox regression models and are presented if they emerged as statistically significant (p<0.05). *LA Es, LA EF and LAS/LAS_90_ were considered in separate multivariate models due to their high correlation (model 1-4). CI = confidence interval, IS = infarct size, MVO = microvascular obstruction, MSI = myocardial salvage index, LV EF = left ventricular ejection fraction, GLS = global longitudinal strain, Es = reservoir function, LAS = long axis strain. | | | | |

NSTEMI

| **Table S3 Univariate Predictors of MACE in NSTEMI** | | |
| --- | --- | --- |
| Variable | Univariable  Hazard Ratio (CI) | p |
| **Cardiovascular/clinical risk factors** | | |
| Age | 1.05 (1.02-1.08) | **0.001** |
| Male Sex | 1.01 (0.50-2.01) | 0.987 |
| Smoking | 0.89 (0.46-1.70) | 0.713 |
| Hypertension | 1.35 (0.60-3.04) | 0.473 |
| HLP | 1.33 (0.72-2.46) | 0.366 |
| Diabetes | 2.67 (1.45-4.92) | **0.002** |
| BMI | 1.06 (1.00-1.13) | **0.042** |
| Killip-Class | 2.88 (1.90-4.37) | **<0.001** |
| Atrial fibrillation | 2.77 (0.83-9.27) | 0.097 |
| Mitral regurgitation | 2.78 (0.82-0.40) | 0.100 |
| **Angiography** | | |
| Diseased Vessels | 1.46 (1.00-2.14) | **0.049** |
| Culprit lesion | 1.48 (1.07-2.04) | **0.018** |
| TIMI pre | 1.08 (0.84-1.39) | 0.563 |
| TIMI post | 0.69 (0.48–0.99) | **0.042** |
| **CMR-derived morphology** | | |
| IS | 1.00 (0.99-1.01) | 0.913 |
| MVO | 1.05 (0.90-1.22) | 0.570 |
| AAR | 1.03 (0.98-1.08) | 0.250 |
| MSI | 1.00 (0.98-1.01) | 0.637 |
| LAVI | 1.03 (1.01-1.05) | **<0.001** |
| **CMR-derived function** | | |
| LV EF | 0.95 (0.92-0.98) | **0.004** |
| LV GLS | 1.13 (1.06-1.21) | **<0.001** |
| LA Es | 0.91 (0.86-0.97) | **0.002** |
| LA EF | 0.95 (0.92-0.98) | **0.001** |
| LA LAS | 0.87 (0.81-0.93) | **<0.001** |
| LA LAS_90_ | 0.87 (0.81-0.93) | **<0.001** |
| The table reports univariable Cox regression models to predict a major adverse clinical event during the 12 months follow-up period following acute myocardial infarction in NSTEMI patients. The data is presented as hazard ratios with associated 95% confidence intervals in parentheses. CI = confidence interval, HLP =Hyperlipoproteinemia, BMI = body mass index, TIMI = Thrombolysis In Myocardial Infarction grade pre/post PCI, PCI = percutaneous coronary intervention, IS = infarct size, MVO = microvascular obstruction, AAR = area at risk, MSI = myocardial salvage index, LAVI = left atrial volume index, LV EF = left ventricular ejection fraction, GLS = global longitudinal strain, Es = reservoir function, LAS = long axis strain. | | |

| **Table S4 Multivariate Predictors of MACE in NSTEMI** | | | | |
| --- | --- | --- | --- | --- |
| Variable | 1. Multivariate Hazard Ratio (CI) | 2. Multivariate Hazard Ratio (CI) | 3. Multivariate Hazard Ratio (CI) | 4. Multivariate Hazard Ratio (CI) |
| **Cardiovascular/clinical risk factors** | | | | |
| Age |  |  |  |  |
| Diabetes |  |  |  |  |
| BMI |  |  |  |  |
| Killip-Class | 2.35 (1.05-5.26)  p=0.037 | 2.29 (1.02-5.13)  p=0.044 | 2.28 (1.02-5.10)  p=0.044 | 2.33 (1.05-5.13)  p=0.037 |
| **Angiography** | | | | |
| Diseased Vessels |  |  |  |  |
| Culprit Lesion |  |  |  |  |
| TIMI post |  |  |  |  |
| **CMR-derived morphology** | | | | |
| LAVI |  |  |  |  |
| **CMR-derived function** | | | | |
| LV EF |  |  |  |  |
| LV GLS | 1.10 (1.01-1.19)  p=0.027 | 1.10 (1.02-1.20)  p=0.016 | 1.10 (1.01-1.20)  p=0.022 | 1.10 (1.01-1.19)  p=0.024 |
| 1 LA Es |  |  |  |  |
| 2 LA EF |  |  |  |  |
| 3 LA LAS |  |  |  |  |
| 4 LA LAS_90_ |  |  |  |  |
| The table reports multivariable Cox regression models (based on the enter method) to predict a major adverse clinical event during the 12 months follow-up period following acute myocardial infarction in NSTEMI patients. The data is presented as hazard ratios with associated 95% confidence intervals in parentheses. Variables with univariate significance (p<0.05) were included in multivariable Cox regression models and are presented if they emerged as statistically significant (p<0.05). *LA Es, LA EF and LAS/LAS_90_ were considered in separate multivariate models due to their high correlation (model 1-4). CI = confidence interval, BMI = body mass index, TIMI = Thrombolysis In Myocardial Infarction grade post percutaneous coronary intervention, LAVI = left atriale volume index, LV EF = left ventricular ejection fraction, GLS = global longitudinal strain, Es = reservoir function, LAS = long axis strain. | | | | |

| **Table S5 Selected Multivariate Predictors of MACE** | | | | |
| --- | --- | --- | --- | --- |
| Variable | 1. Multivariate Hazard Ratio (CI) | 2. Multivariate Hazard Ratio (CI) | 3. Multivariate Hazard Ratio (CI) | 4. Multivariate Hazard Ratio (CI) |
| **Cardiovascular/clinical risk factors** | | | | |
| Age | 1.03 (1.00-1.05)  p=0.025 |  |  |  |
| Killip-Class | 1.43 (1.06-1.94)  p=0.019 | 1.43 (1.06-1.93)  p=0.021 | 1.42 (1.05-1.91)  p=0.022 | 1.43 (1.06-1.93)  p=0.021 |
| **Angiography** | | | | |
| Diseased Vessels |  |  |  |  |
| **CMR-derived morphology** | | | | |
| IS |  |  |  |  |
| MVO |  |  |  |  |
| **CMR-derived function** | | | | |
| LV EF |  |  |  |  |
| LV GLS | 1.09 (1.03-1.17)  p=0.006 | 1.10 (1.03-1.17)  p=0.003 | 1.09 (1.02-1.16)  p=0.011 | 1.09 (1.03-1.16)  p=0.006 |
| 1 LA Es |  |  |  |  |
| 2 LA EF |  |  |  |  |
| 3 LA LAS |  |  | 0.91 (0.85-0.98)  p=0.007 |  |
| 4 LA LAS_90_ |  |  |  | 0.92 (0.87-0.98)  p=0.007 |
| The table reports multivariable Cox regression models (based on the enter method) to predict a major adverse clinical event during the 12 months follow-up period following acute myocardial infarction. The data is presented as hazard ratios with associated 95% confidence intervals in parentheses. The eight variables with highest significance in univariate significance were included in multivariable Cox regression models and are presented if they emerged as statistically significant (p<0.05). *LA Es, LA EF and LAS/LAS_90_ were considered in separate multivariate models due to their high correlation (model 1-4). CI = confidence interval, IS = infarct size, MVO = microvascular obstruction, LV EF = left ventricular ejection fraction, GLS = global longitudinal strain, Es = reservoir function, LAS = long axis strain. | | | | |


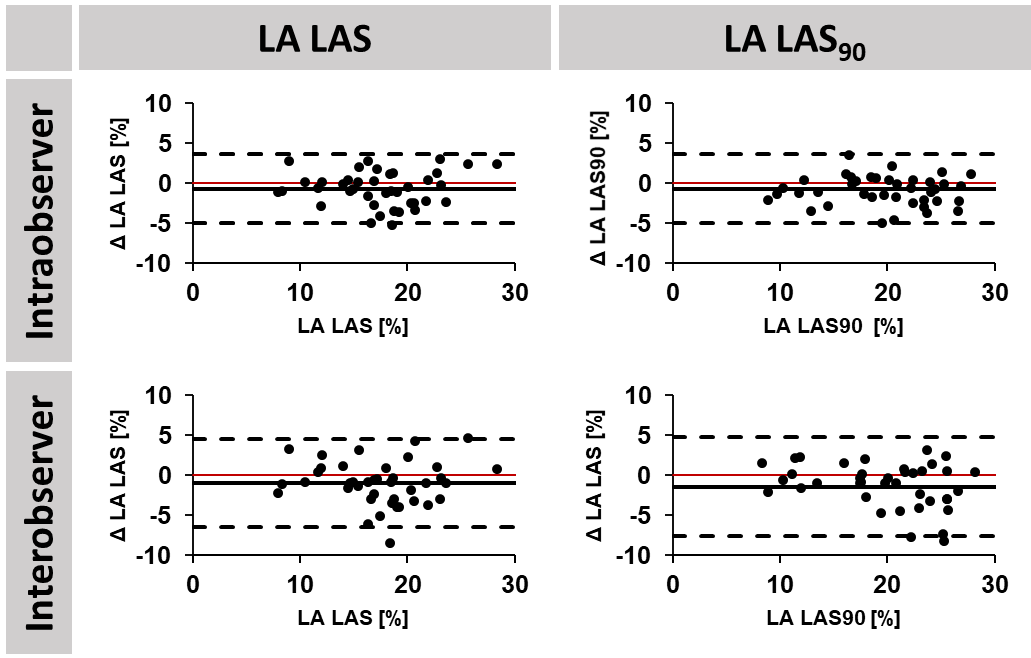


**Figure S1 Reproducibility**

Bland Altman plots are shown for left atrial (LA) long axis strain (LAS) obtained by the method of the longest distance as well as the perpendicular approach. Δ: difference readout one and two. Reference for 0 difference in red.
